# Recycling of Agricultural Film Wastes for Use as a Binder in Building Composites

**DOI:** 10.3390/ma18020251

**Published:** 2025-01-08

**Authors:** Bartosz Zegardło, Chrysanthos Maraveas, Kacper Rastawicki, Paweł Woliński, Antoni Bombik

**Affiliations:** 1 Research Team of Quantitative Methods and Spatial Management, Institute of Agriculture and Horticulture, Faculty of Agricultural Sciences, University of Siedlce, B. Prusa 14, 08-110 Siedlce, Poland; kr82729@stud.uph.edu.pl (K.R.);; 2 Department of Natural Resources and Agricultural Engineering, Agricultural University of Athens, Leof. Athinon 51, 104 47 Athens, Greece; c.maraveas@maraveas.gr; 3 Department of Construction, Faculty of Technical Sciences, Academy of Applied Sciences Mazovia, Sokołowska 161, 08-110 Siedlce, Poland

**Keywords:** polyethylene, LDPE, recycling, green composites, plastic wastes, agricultural wastes, green concretes, building sector

## Abstract

Plastic film, also known as low-density polyethylene (LDPE), poses serious environmental challenges due to mass production, short life cycle, and poor waste management. The main aim of this paper was to examine the suitability of using agricultural waste film as a binder in construction composites instead of the traditional cement slurry. Molten at temperatures of around 120–150 °C wastes was mixed with fine sand and gravel aggregate as filler. Twelve samples consisting of different mixtures were produced—F20, F25, F30, F35, F40, F45, F50, F60, F70, F80, F90, and F100—where a given number indicates the weight ratio of film waste to aggregate used. The composites were subjected to various tests, including volumetric density, compressive strength, and flexural strength. The volumetric density (ρ) of the composites decreased with increasing amounts of waste. Composites containing 100% recyclate (F100) depicted density, ρ = 0.74 g/cm^3^, was 50.7% lower than for a composite that contained 20% recyclate (F20). The highest soakability was recorded in F20 (2.19%). Subsequently, the absorbency tested in composites decreased with increasing recyclate content. Compression strength (σ_comp_) was highest for F40 (σ_comp_ = 39.46 MPa). In contrast, F20 had the lowest recorded compressive strength value (σ_comp_ = 11.13 MPa) and was 71.8% lower than F40. F70 had the highest recorded flexural strength value (σ_flex_ = 27.77 MPa). The other composites showed lower strength for higher amounts of recyclate and the amount of sand. SEM imaging proved that the contact zone between the aggregate grains and the bonding phase of the recycled film was consistent, with no anomalies, cracks, or voids. The results prove that LDPE film waste is suitable for use as a binder in building composites. However, appropriately selecting proportions of the recyclate, sand, and gravel aggregate is crucial to obtain a composite with technical parameters similar to those of cementitious composites.

## 1. Introduction

Most packaging materials, including reusable bags, are made from plastics due to their lightness, flexibility, and good water-repellent properties. The most commonly used polymer for packaging products is low-density polyethylene (LDPE), which presents the additional advantage of a low price/satisfactory product quality ratio [1]. LDPE is a flexible, waxy, transparent, thermoplastic [2] that loses its elastic properties after prolonged exposure to sunlight and moisture. Polyethylene films have low permeability to water vapour, are resistant to acids and alkalis, and can withstand low temperatures, making them a preferred option for packaging applications [3]. According to the European Plastics Manufacturers’ Association Plastics Europe, global plastics production in 2022 was 390 million tonnes, approximately 4% higher than the previous year [4]. In 2020, the annual demand of the European Union alone for LDPE was 8.85 Mt [5]. Despite the continuous increase in effective recycling of film packaging in EU countries, the rate is still low, around 20%, and looking at the economies of all countries in the world, the global recycling rate of this waste is even lower, around 9% [5]. Post-consumer recycling of LDPE is problematic, leading to large amounts of the plastic being disposed in landfills and incinerated, thereby contributing to soil, water, and air pollution [6]. Considering the environmental challenges of LDPE and the desire to meet the growing demand for the product [7], it is necessary to adopt sustainable measures of using waste plastics to minimise negative environmental impacts [8]. In the last two decades, there has been an emphasis on a circular economy in most countries to promote recycling, reuse, and sharing of resources to minimize environmental pollution [9] The circular economy model is also considered crucial in addressing issues related to ecosystem changes, climate change, water shortages and loss of biodiversity [10]. The strategy should particularly target sectors with unsustainable practices, such as high exploitation of natural resources, the pollution of water and air by chemical compounds and heat during production processes, and the accumulation of waste on land rendered biologically inactive by this fact [11,12,13]. The proposed solution of a closed-loop economy [14] entails 100% of manufactured and end-of-life products being used to manufacture the same new products [15] or other products in which waste would constitute the production substrate. Extensive research has been conducted in this area, and has revealed different technologies that can support a circular economy. A basic division of current technologies for the treatment of artificial waste divides it into four categories [16]: primary (blown sleeve re-extrusion), secondary (mechanical), tertiary (chemical recycling), and quaternary (energy recovery).

Primary recycling involves the reintroduction of pure polymer scrap into the production cycle to make products from the same material. The approach is commonly used only on the process line itself to capture in-plant waste [17]. Although primary recycling is relatively simple due to the purity and homogeneity of the waste, it is rarely used by recyclers taking products from the waste market due to quality concerns of the recycled product. Interestingly, re-extrusion is often overlooked as a recycling technology because the reused material never reaches the consumer and is not exploited by the consumer before being recovered [18].

Mechanical recycling, also called thermal recycling, is a common method of plastics recycling. The technique consists of several processes: segregation, cleaning the waste, shredding, and thermal processing—melting at high temperatures and granulation. The granules, flakes, or powders thus formed are used as a substrate for the remanufacturing of products by either sleeve extrusion or by pouring, a process called flat-slot extrusion. Existing studies indicate that thermal recycling method has a negligible effect on the molecular weight of the plastics [19] and on their mechanical properties [20]. Due to the available well-described procedures and known processing technology, this recycling method is widespread [21]. The main concern in using the technique is not remelting, but careful product separation (including colour) and thorough cleaning, which are energy- and cost-intensive processes.

The third recycling method observed in economic activities to which other plastics are also subjected [22,23,24,25] is chemical recycling [26,27], also called advanced recycling [28,29,30,31]. In this process, pure compounds are obtained from plastics for the production of polymers and in turn new products. Recycled polymers are either depolymerised into their original components and repolymerised into a new oligomer or solvated (solvolysis) to dissolve the polymers and subsequent purification [32]. For various plastics, the polymer chain is either partially broken down into smaller oligomers or completely broken down into monomer units, liquids, and gases [33]. Chemical recycling is implemented using different methods. The first, less common, is methanolysis [34,35], in which plastics are depolymerised by reaction with methanol at high pressure (2–4 MPa) and temperature (180–280 °C) [36]. Another method is glycolysis, the most economical and commercially used approach for plastics chemical recycling. Glycol is used to break down ester bonds, and the reaction takes place under pressure, with temperatures widely ranging from 245 °C (catalysed glycolysis) [37], 450 °C (supercritical glycolysis) [38], to 170–175 °C (microwave-assisted catalysis) [39]. Other less well-known chemical method is hydrolysis that is conducted at high pressures of 1.4–2 MPa and requires temperatures of 200–250 °C [40,41] under acidic, basic, or neutral conditions, and amino lysis [42,43]. The recycling approach also requires thorough waste segregation and treatment. However, the aforementioned chemical and thermal processes present relatively high costs that discourage product manufacturers to consider them when producing new products from recyclates. Since producers mainly focus on maximizing economic profits, they often opt to purchase production substrates such as oil and gas derivatives extracted directly from natural deposits.

Additionally, an energy recovery technique can be used to recycle LDPE plastics [44]. However, some critics consider the technique a waste disposal method instead of recycling, because the system does not return the exchanged matter to recirculation. In the approach, incineration is used to neutralise LDPE and other plastics [45,46]. Therefore, recycling itself can only refer to the reuse of the chemical energy accumulated in polymers that is converted into heat energy. The process is recommended only for contaminated waste, where it is costly and difficult to use mechanical or chemical recycling processes. The main disadvantage of the energy recovery method entails releasing harmful chemical compounds into the air. For this type of waste incineration, emissions of dioxins, sulphur dioxide, nitrogen oxides, hydrogen chloride, mercury, and cadmium are controlled to prevent poisoning risks [47].

Although there are many plastics recycling methods, most LDPE packaging films end up in landfills after single use [48]. The plastic films are not commonly recycled due to their flexibility, low compressive strength, and affordable raw materials. In 2017, over 90 percent of manufactured products globally were created from fossil fuels [49]. The trend underlines the important role of plastics in modern society. However, one challenge that discourages recycling of plastic waste is the costly process of sorting various plastic waste streams, making recycling uneconomical for LDPE. Therefore, it is likely that packaging waste will continue to heavily pollute the environment unless efficient strategies of separating plastics are developed.

The rationale of the present study was to address the LDPE recycling challenges by using methods that do not require high purification thresholds, are relatively inexpensive, and economically viable. For the present study, the use of LDPE packaging waste in the construction industry was identified as an alternative to traditional recycling techniques.

The construction industry is one of the most rapidly growing sectors of the economy in which a wide range of recycling options can be implemented [50,51,52,53]. In developed countries, there are increased calls for actions to manufacture new products from recyclates, while ensuring the technical parameters are similar to products manufactured using traditional methods [54]. Extensive research work in the scientific community [55,56,57,58] points to the construction composite industry as a potential place for managing large amounts of waste [59,60,61,62,63,64]. The use of different wastes in composite manufacturing helps to neutralise huge deposits of unwanted matter while helping to meet the huge demand of affordable construction products [65,66,67]. However, wastes are only effective as recyclates if they are non-biodegradable, [68,69,70,71,72,73], and hence cannot compromise structural integrity of buildings where they are used. The right choice of recyclate for composites allows the disposal of unwanted matter, but should also enhance the technical performance of the new product. The idea has been highlighted in many research papers, including those in which recyclates improve abrasion resistance [74] of building composites and allow ultra-high strength [75], high temperature resistance [76], high chemical resistance [77], heat storage capacity [78] or resistance to sewage effluent environments [79]. Many authors agree that the construction industry provides hope for waste management [80,81,82], and the use of recyclates in an optimal way [83,84] makes it possible to obtain composites with characteristics superior to products made from traditional substrates.

Several studies have been conducted on the use of plastic waste as a component of building composites. The main conclusions from these works, relating to the basic technical parameters to which traditional building composites are subjected [85,86,87,88], is that an increase in the volume ratio of plastic waste leads to a decrease in the composite mixture’s workability [89,90,91,92,93,94,95]. In some cases, adding 15% recyclate resulted in a 40% lower workability [96]. Adding plastic to composites leads to a decrease in bulk density [97,98,99,100,101]. The trend is related to the fact that traditional composite components like cement or aggregates used in the production of traditional composites have a much higher volumetric weight than plastic recyclates. Specifically, Ismail and Al-Hashmi [102] tested samples containing 10%, 15%, and 20% plastic waste in the volume of the building composite, and found that the density of the composite decreased by 5% and 7%, respectively. The authors also proved a certain regularity regarding the absorbability of composites [102]. The greater the amount of additive used, the lower the absorbability of the composites. Meena et al. [103] found that low absorbability is associated with positive consequences, since a low-absorbance material is more resistant to environmental factors, such as freeze–thaw cycles of potentially contained water. Therefore, the low absorbability feature t increases composite durability [104,105]. In contrast, the available research shows reduced strength of composites made from plastic recyclates, including compressive strength, tensile strength, modulus of elasticity, and unit weight [106,107]. In particular, Pereira et al. [108] demonstrated that recyclate volume depicts an inverse relationship with composite compressive strength. This conclusion is expected, as cement, stone, and fine aggregates are materials with a much higher compressive strength than plastic recyclate. However, literature evidence also shows cases where adding recyclate increases composite tensile strength [109,110,111,112,113], which is a low parameter for traditional components, especially in cementitious composites. As can be seen from the above examples, the use of waste plastics is potentially possible and has great prospects. In the future, it is also worth highlighting research at small scales, e.g., strategies to increase interfacial adhesion properties between materials to produce composites with improved properties at the nanoscale [114] and how this knowledge can support the development of new smart materials suitable for the construction sector [115].

Considering this backdrop, the present study focused on investigating the suitability of building composites made from LDPE recyclates that substitute the traditional cement slurry. After coarse cleaning, foil waste was melted at 120–150 °C in an open vessel located on a gas burner and used as a binder in this melted form together with traditional sand and gravel aggregates. The research hypotheses assumed the possibility of obtaining composites with technical parameters at least equal to those of cementitious composites used to make structural elements of buildings.

## 2. Materials and Methods

### 2.1. Materials

Several materials were used as substrates in the experiment: (i) LDPE film waste and (ii) aggregates consisting of sand and gravel. Plastic waste was obtained as part of disposed farmhouse wastes and transported to the laboratory. The first step involved cleaning the waste to remove dirt and then separating other solid materials from plastic. The film prepared in this way was cut into fragments no larger than 20 × 20 cm to make it easier to melt and mix it with aggregate and to properly weigh out the required recyclate amount. The basic technical parameters of the film taken from the manufacturer’s data are presented in Table 1.

Sand and gravel aggregates were obtained from quarries located in eastern Poland and used as the second component of the composite. Only fine aggregate with a grain fraction less than 2 mm was separated and used in the mix after applying the sieve analysis method. Table 2 presents sand and gravel aggregate basic technical parameters based on manufacturers’ data.

The composite manufacturing strategy process followed a four-step scheme:

(1) Determination of the melting point of the recyclate.
(2) Evaluation of the maximum aggregate saturation of the melted recyclate.
(3) Preparation of a weighting recipe for the composite with maximum aggregate saturation of the recyclate.
(4) Preparation of further weight prescriptions for the other composites with gradually decreasing aggregate saturation.

The first stage involved temperature tests during component mixing. Preparing the composites involved heating the weighted sand to a temperature of 100 °C in a vessel over a gas burner fed from a cylinder filled with propane–butane gas. The previously prepared foil waste was gradually added to the heated aggregate. The foil, when exposed to slightly higher temperatures of around 120–150 °C, shrank and became plastic enough to allow individual grains of aggregate to penetrate the plastic foil structure. The two components were mixed at this temperature until all the intended elements were kneaded to achieve a uniform consistency.

Material quantities were selected iteratively using the successive approximation method. The previously weighed recyclate was mixed with the prepared and weighed portion of sand. Once these components were mixed, heated sand was again added to the mixture until the maximum aggregate saturation was reached, at which point the sand grains were surrounded by the thinnest possible layer of molten waste. The results of these tests revealed that the maximum weight ratio of sand to recyclate at which the sand grains are bonded and in direct contact with each other was 2:8. A laboratory test was also conducted on the prepared mixture, where the mixture volume was measured. Based on the trial mixture weight–volume measurements and theoretical densities of the described components in the spreadsheet, the components were converted to the composite recipe, taking into account the weights of the substrates per 1 m^3^ of the ready-mixed composite. The calculations carried out during the iterative selection of components for the composite containing 20% by weight of LDPE recyclate and 80% by weight of aggregate are presented in Table 3. This scheme of composing the components of the composite was implemented for all planned mixes.

Twelve sample mixtures were prepared—F20, F25, F30, F35, F40, F45, F50, F60, F70, F80, F90, and F100—in which a given number indicated the percentage by weight of LDPE film waste material used in relation to the composite mix weight. The recipes of the individual mixes per 1 m^3^ of composite mix are presented in Table 4.

**Figure 1:**
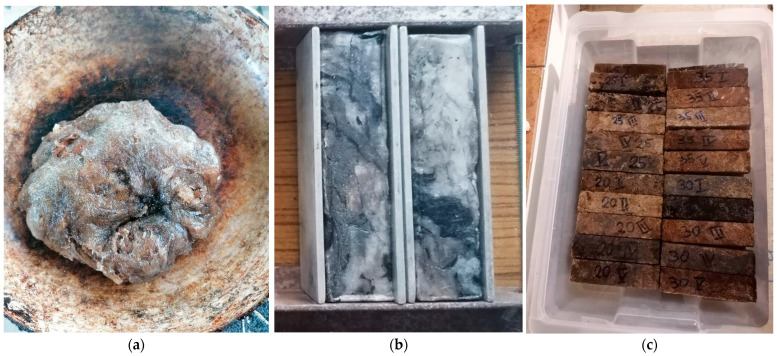
Composite samples using LDPE low-density polyethylene film waste as a binder for sand and gravel aggregates. (**a**) Mixing of rock in a vessel on a gas burner; (**b**) F100 samples without aggregate in the prepared moulds; (**c**) F20–F35 composite samples containing 20–35% by weight of sand and gravel aggregate, respectively (clear difference in colour of samples due to visibility of aggregate).

Figure 1 shows the composite samples during their preparation (a) in a pot on a gas burner, (b) in moulds, and (c) prepared for the soak test.

### 2.2. Testing Procedures

Implementing the planned research involved conducting basic tests similar to those done on conventional construction materials.

All samples were prepared in the same way. In the first phase, cement slurry and PET fibres were prepared in separate containers. Successive portions of the waste material were added to the slurry and mixed in a rotary mixer. Ten samples of each test series were made.

All prepared concrete mixtures were evaluated for consistency, also known as the standard test according to PN-EN 12350-2:2011 [116] on testing concrete mixture—Part 2: consistency testing used the cone drop method.

Volumetric density was tested on cubic samples measuring 4 × 4 × 16 cm. The samples were measured by an OWL meter and weighed on the SILVER scale as per EN 12390-7:2011 [117]. The volumetric density was calculated as the ratio of the volume of the tested samples to their weight. The analyses used the formula:

ρ = m/V

where ρ was the specific density (kg/dm^3^), m was the mass of the sample (kg), and V was the volume of the sample (dm^3^).

Water absorption was tested on identical samples as volumetric density. The samples were immersed and remained in the water until their weight was established. The water absorption was calculated according to [118] as the ratio of the amount of water the composite was able to absorb to the weight of the dry composite, expressed as a percentage. The analyses used the formula:

S = m_water_/m_dry_ × 100%

where S was the absorption (in %), m_water_ was the mass of water absorbed by the sample (kg), and m_dry_ was the dry mass of the sample (kg).

The flexural strength of the three-point scheme was tested according to the method highlighted in PN-EN 12390-5:2009 [119]. The analyses used the formula:

$$\rho_{\mathrm{flex}}\;=\;(3\mathrm{FL})/{2\mathrm{bh}}^{2}$$

where σ_flex_ was the flexural strength (MPa), F was the maximum force applied at the midpoint of the sample (N), L was the span length (distance between supports) (mm), b was the width of the sample (mm), and h was the height of the sample (mm).

Moreover, specimens measuring 4 × 4 × 16 cm were prepared for the test, and the compressive strengths of the specimens were tested according to the method in PN-EN 12390-3:2011 [120]. The 4 × 4 × 4 cm specimens were tested after the specimens were broken during the flexural strength test. The analyses used the formula:

σ_comp_ = F/A

where σ_comp_ was the compressive strength (MPa), F was the compressive force applied to the sample (N), and A was the cross-sectional area of the sample (m^2^).

The strength test was conducted on a Matest 2000 testing machine, with a 0–300 kN strain gauge attachment also from Matest (model C089PN468, factory number C089PN468/AA/0001).

The last tests carried out were microscopic studies. Scanning electron microscopy and energy-dispersive X-ray spectroscopy were used in these tests. The composite samples were immersed in resin prior to testing, after which a slow-speed blade was used to cut through the entire prepared test material, showing the internal structure of the composite. Micrograph acquisition was accomplished using Tescan Vega Compact LMH equipment. A secondary electron detector was used for images and an EDAX detector with a Si_3_N_4_ window was used for elemental analysis. The images and elemental analysis were conducted using an EssenceTM suite. The following measuring settings were used during the tests: type: map, profile: resolution, mode: fixed time, counts: 154 983, real time: 301.55, live time: 300.054, dead time: 0%, landing energy: 20 keV, beam current: 100 pA, coating element: gold.

## 3. Research Results and Analysis

In testing the composite mixture consistency with reference to the tests conducted on the concrete composite, all of them had a dense-plastic consistency when heated, which, by reference to the cone drop test, would be assessed as S1 with a drop of between 10 and 90 mm. Mixing of the components was easier when the quantity of aggregates was smaller. A larger amount of sand caused difficulties in distributing it evenly in the mixture. A similar situation was observed when the mixture was placed in the moulds. The mixture with only 20% foil waste was laid in the moulds with a tendency to delaminate. The mix containing only foil waste F100 was laid without delaminating, and its consistency was the most workable of all the mixes.

Comparing this study to others on conventional concretes containing plastic recyclates as fillers, a different result was obtained. Previous researchers who analysed the effect of plastic additive on composite noted that as the volume ratio of plastic waste increased, there was a decrease in concrete workability [85,86]. As the workability of composites and their ease of placement in moulds is a significant characteristic, the proposed waste dosage form was considered to have a positive effect on the analysed characteristic.

**Figure 2:**
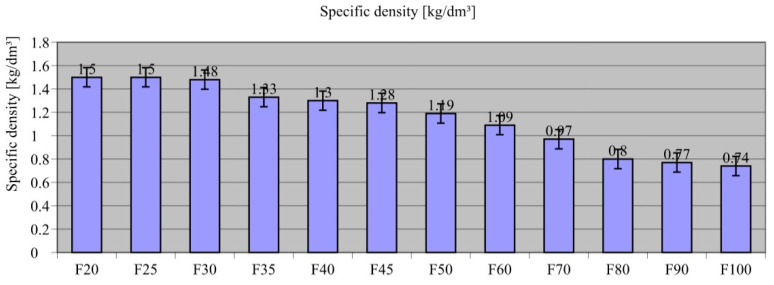
Results of specific density testing of composites.

Figure 2 shows the results of the specific density of the composites.

The assessed specific density of the composites was highest for the composite containing only 20 wt.% waste film F20 (Figure 2). F20 composite density was 1.50 g/cm^3^, similar to F25 (ρ = 1.50 g/cm^3^), for which the recyclate accounted for 25 wt.%, and only 1.33% higher than F30 (ρ = 1.48 g/cm^3^). The bulk density decreased with increasing amount of waste, where it was 48.7% (ρ = 0.77 g/cm^3^) and 50.7% (ρ = 0.74 g/cm^3^) higher for composites containing 90% recyclate (F90) and 100% (F100), respectively. The result was also related to theoretical calculations and to the theoretical values of LDPE recyclate density. The theoretical LDPE density of 0.92 g/cm^3^ was higher than that tested for samples containing recyclate alone, and the densities measured for samples with a high recyclate content were also lower than the theoretical values. A conclusion was drawn that the samples containing LDPE waste also contained air, which may have remained confined during the mixing and moulding process. The trend was confirmed by the finding of a decline in composite density as more recyclates were used to produce the composite.

The obtained study findings also confirmed those of other researchers [98,100,101], highlighting that since the waste material has a much lower density (ρ = 0.92 g/cm^3^) than sand aggregate (ρ = 1.78 g/cm^3^), the addition of film waste had a lowering effect on the density of the composite. Similarity was also noted after comparing the numerical values obtained in this research to the work of other authors. Ismail and Al-Hashmi [102] used plastic recyclate as a substitute for aggregate in composite production, with its content reaching 20% of its weight, and noted that the composite density decreased by 8.7%. In the present study, the difference in composite density between F20 (ρ = 1.50 g/cm^3^) and F40 (ρ = 1.30 g/cm^3^) for which the recyclate content was 20%—the difference in bulk density was 13.3%. Therefore, the trend observed was relatively similar, and the discrepancies may have been due to the form of the recyclates used.

**Figure 3:**
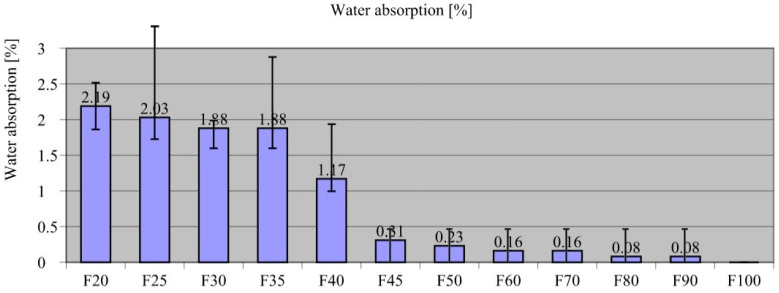
Results of the saturation test of the composites.

Figure 3 shows the results of the saturation test on the composites.

The highest absorbency was recorded in F20, at 2.19%. Subsequently, the results indicated decreasing absorbability for composites with increasing recyclate content. The absorption of F25 composite was lower than F20 by only 7.3% (2.03%), but for F90, it was lower by as much as 96.3% (0.08%). There was no recorded absorption for F100. The absorption test results were aligned with the volumetric density test, leading to the assumption that if there are air pores within the composite sample, they are airtight and not accessible from the outside. Another interesting finding in this study was the rather large scatter of results observed for composite samples with high aggregate content (F20–F35). This was indicative of the rather high non-uniformity of this characteristic within this group of composites. Sand aggregate is an absorbent aggregate, while recyclate is a zero-absorbance material. Therefore, composite absorbability decreased significantly as the amount of recyclate added increased. Similar observations were made by other researchers describing construction composites containing plastic recyclates, where they highlighted adding plastics could improve the durability of building composites [104,105]. Expressly, Meena et al. [103] pinpointed that the lower absorbability of composites significantly affects their durability, especially under cyclic freezing and thawing conditions. Nonetheless, it is destructive to increase the volume of water in the composite capillaries, which can cause it to crack and fail. Therefore, it can be concluded that the proposed method of recycling film waste can produce a building composite that is not susceptible to this phenomenon. It is also worth noting that long-term exposure to water did not change the structure of the composites, and no swelling or other adverse processes were observed, which is important in terms of building materials exposed to constant water exposure.

**Figure 4:**
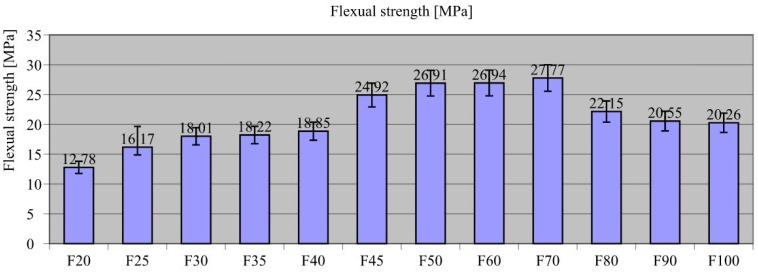
Flexural strength results of the composites.

Figure 4 presents the results of flexural strength tests on the composites.

As shown in Figure 4, F70 composite had the highest recorded flexural strength (σ_flex_ = 27.77 MPa) and was considered optimal in terms of this parameter. The other composites had lower flexural strengths for higher amounts of sand and recyclate. Specifically, the flexural strength for F20 (σ_flex_ = 12.78 MPa) was as much as 53.9% lower than F70. Similarly, for composites containing a higher amount of the additive, the value of this parameter gradually decreased. For composites F80, F90, and F100, the values were lower by 20.2% (σ_flex_ = 22.15 MPa), 25.9% (σ_flex_ = 20.55 MPa), and 27.1% (σ_flex_ = 20.26 MPa), respectively.

**Figure 5:**
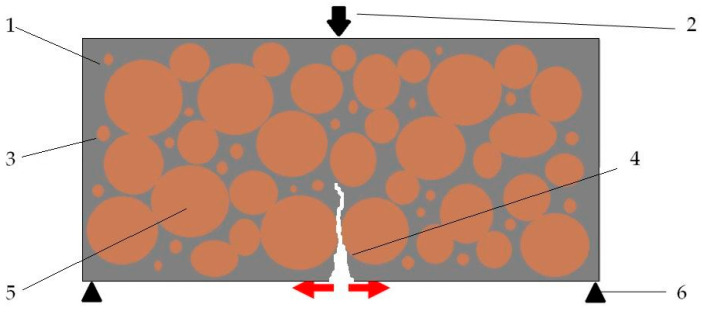
Diagram of the phenomenon of negative aggregate overfilling in the composite. In the tensile zone (4), the contacting aggregate between which there is insufficient filler cannot transmit tensile stresses. Propagating scratching of the test specimen then occurs. Designations: (1)—bonding phase with LDPE recyclate, (2)—bending force, (3)—fine aggregate, (4)—local tensile stresses, (5)—coarse aggregate grains, (6)—supports of the bending specimen.

The test results showing a decrease in composite flexural strength with a greater amount of sand are related to observations regarding the consistency of the mixture and its formability. The composite samples with high sand content and a low binder phase content could hypothetically have had zones in their volume, not seen during the preparation of the test mixes, in which the binder did not fill all the free spaces between the aggregate grains, and these grains were in contact with each other and had an effect on the delamination of the composite observed at the time when it was laid in the moulds. This trend means that the tensile stresses (Figure 5(4)) between the grains in contact with each other could not be properly transferred.

In these areas, there was likely weakening of the composite affecting both compressive and tensile strengths. However, results showing the decrease in composite tensile strength with increasing amount of recyclate above a certain level could not be effectively explained in this study. The tensile strength of the waste film material is approximately 14 MPa. By relating this tensile strength value to F100 flexural strength (σ_flex_ = 20.26 MPa), it is concluded that the remelting of the foil waste strengthens the structure of the composite itself and causes an increase in this parameter. The tensile strength of the gravel aggregate is approximately 40 MPa. Therefore, it is likely that the aggregate itself and the strong bond between the aggregate and the recyclate could have made the value of this parameter for the optimum composition of the F70 composite the highest.

Relating this result to that of other researchers [102,107] confirmed the observation that using plastic recyclates as substrates for building composites is justified and has a positive effect on this technical parameter. The studies presented in the above-mentioned work showed that the flexural strength of the composite sometimes increases with an increase in the proportion of recyclate. In the work of Belmokaddem et al. [107], they found that the introduction of plastic waste as an aggregate replacement in concrete can lead to an increase in tensile strength at low replacement levels. In the study, it was observed that at 5% replacement of coarse aggregate with plastic waste, the tensile strength increased by 16% compared to the control sample, reaching a value of 2.45 MPa compared to 2.1 MPa for the reference sample. Ismail and Al-Hashmi [102] reported an increase in tensile strength in the range of 10–15% by weight replacement of sand with plastic waste. For 10% replacement, the tensile strength increased by about 6% compared to the reference concrete, and for 15% replacement, the increase in tensile strength was about 8%.

**Figure 6:**
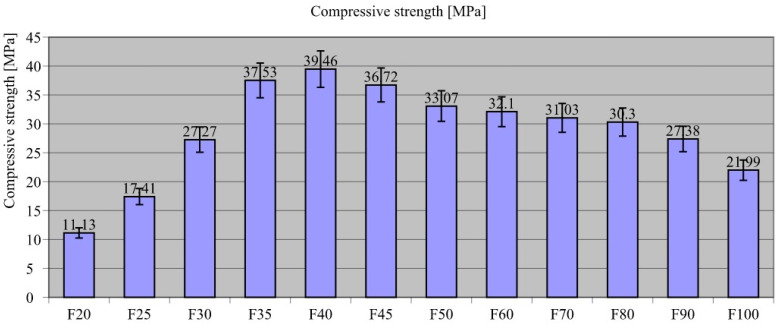
Compressive strength results of the composites.

Figure 6 presents the results of compression strength tests on the composites.

The composite compressive strength test results showed that F40 exhibited the highest value (σ_comp_ = 39.46 Mpa). On this basis, it was concluded that a ratio of film waste to sand filler of 4:6 is needed for optimal composite compressive strength. The trend in Figure 6 shows that increasing recyclates proportion in composite led to a corresponding increase in compressive strength up to the 40% level, beyond which it led to a steady decline in compressive strength. For F20, the lowest compressive strength value was recorded (σ_comp_ = 11.13 MPa), and was as much as 71.8% lower than F40 composite value. Similarly, F90 and F100 had strengths 30.6% (σ_comp_ = 27.38 MPa) and 44.3% (σ_comp_ = 21.99 MPa) lower than F40, respectively.

**Figure 7:**
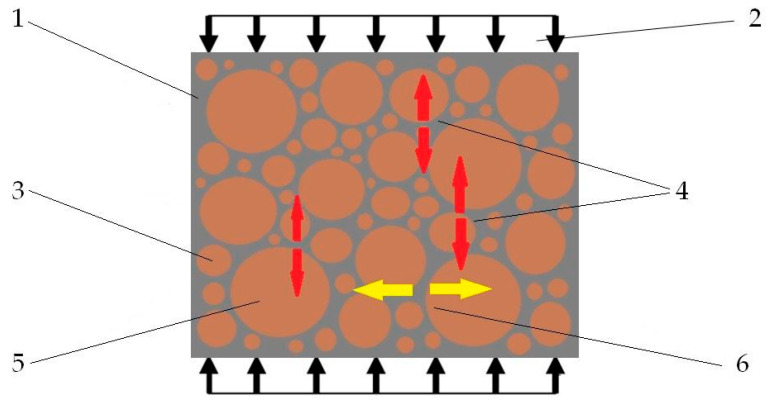
Diagram of the phenomenon of compressive load transfer through the aggregate crumb stack. The aggregate grains interact with each other to transfer compressive forces (4). Local grain-to-grain pressures result in local tensile stresses in the bonding phase of the composite (6). Designations: (1)—LDPE recycled binder phase, (2)—compressive force, (3)—fine aggregate, (4)—local compressive stresses, (5)—coarse aggregate grains, (6)—local tensile stresses.

In explaining the study results, reference was made to the essence of introducing aggregate into building composites. Despite the fact that aggregate is an economically viable filler, it also plays a critical role in the composite load transfer process. A properly designed composite should be filled with aggregate of different granulations so that the fine grains tightly fill the spaces between the coarser aggregate. The total proportion of aggregate in the composite (fine and medium combined) should be between 30% and 60% of the weight of the composite, with fine aggregate (up to 2 mm) making up about 50–60% of the total aggregate in the composite. This aggregate fills the spaces between the larger grains, improving tightness and strength. Medium aggregate (2 mm–4 mm) should make up about 40–50% of the total weight of the aggregate. It works with the finer grains to provide the right structure and optimum performance of the composite. Such a tightly packed aggregate framework transfers compressive stresses to the subsequent layers of the composite (Figure 7(4)). The main function of the binder material in the composite involves surrounding the aggregate grains and fully bonding them together, enabling the transfer of local tensile stresses arising from the interaction of the aggregate grains (Figure 7(6)).

Therefore, the study of this parameter indicated that for this type of composite, the optimum ratio for achieving high compressive strength, which already classifies the composite into structural concretes, is a recyclate–aggregate weight ratio of 4:6. The obtained result proved the validity of using a combination of aggregates with high compressive strength and a binder made from LDPE recyclate with high tensile strength.

Comparing the present study results with those in the literature on cementitious composites and the addition of plastic fillers to them [106,107], the obtained findings somewhat confirmed those noted by previous researchers. The noticeable trend towards an increase in the addition for F50–F100 composites proved that compressive strength decreased as the amount of recyclate in composites increased. Identical observations were described by Pereira et al. [108], in which the compressive strength decreased with the addition of recyclate.

The last tests conducted were microscopic studies, in which scanning electron microscopy and energy-dispersive X-ray spectroscopy were used. The composite samples were immersed in resin before testing, followed by cutting through the entire prepared test material using a slow-speed blade to show the composite internal structure. The acquisition of the micrographs was accomplished utilising the Tescan Vega Compact LMH. A secondary electron detector was used for imaging and an EDAX detector with Si_3_N_4_ window was used for elemental analysis. The EssenceTM suite was used to analyse images and elemental aspects of the composites.

**Figure 8:**
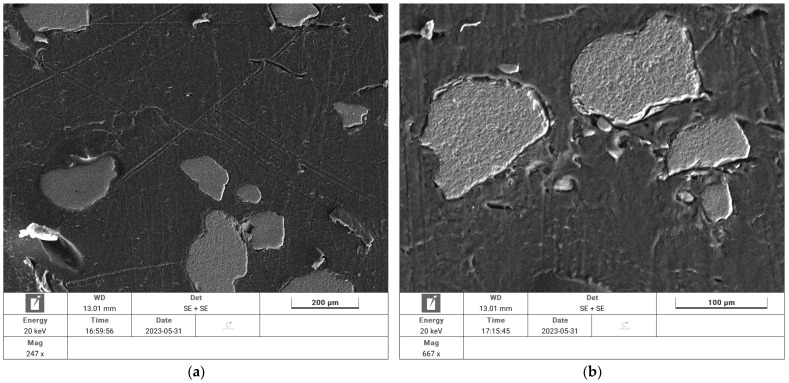
SEM images of a building composite containing LDPE film waste as the bonding phase: (**a**)—view of aggregate grains surrounded by the bonding phase from the recyclate, (**b**)—close-up of the bonding phase–aggregate grain interface.

Figure 8 presents the images obtained during the analyses.

The photographs show details of aggregate grains and the binder made from LDPE film recyclates. Close-ups show that the binder adheres tightly to the aggregate grains and there are no spaces between them. The contact zone is full enough to also fill the cavities in the irregularities of the aggregates. The image proves that the bond strength with the LDPE binder is high and durable, especially due to the development of the contact zone in the aforementioned hollows of the aggregate grains.

**Figure 9:**
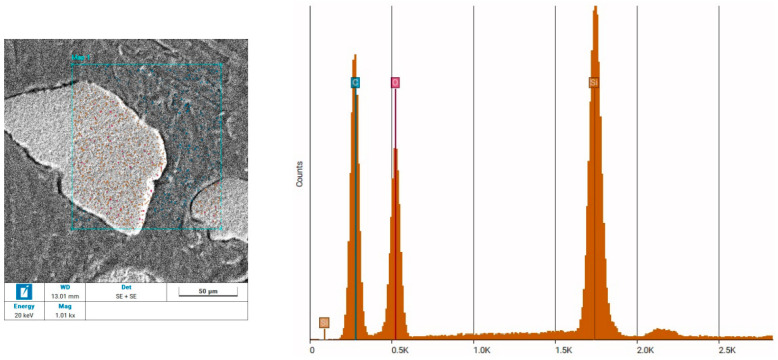
Results of an elemental composition study performed using energy-dispersive X-ray spectroscopy.

An analysis of the elemental composition of the zones highlighted in the photographs was also conducted to confirm the conclusions from the microscopic images. The obtained results of elemental composition are presented in Figure 9.

The elemental composition analysis of the zone as a binder and an aggregate confirmed the validity of the identified images. The main component of the aggregate, shown as brown grains in the image, was Si, which was interpreted as the sand grains from which this aggregate is made. The zone marked with blue grains showed that the main component was C, which was consistent with the elemental composition of the binding phase of the LDPE recyclate—(CH_2_–CH_2_)n.

## 4. Conclusions

The research findings showed that after cleaning agricultural film waste, grinding, and mixing it with sand and gravel aggregates at 150 °C, the obtained composites had varying technical parameters depending on the component proportions used in their production. The highest volumetric density value was recorded for F20 (ρ = 1.50 g/cm^3^), and was 1.33% higher than F30 (ρ = 1.48 g/cm^3^). The bulk density decreased with increasing recyclate. Compared to F20, composites containing 90% recyclate (F90) and 100% (F100) showed 48.7% (ρ = 0.77 g/cm^3^) and 50.7% (ρ = 0.74 g/cm^3^) lower density, respectively. Additionally, F20 recorded the highest absorbency of 2.19%. Subsequently, there was a decrease in absorbability of composites with increasing recyclate content. Specifically, absorbability was 7.3% (2.03%) lower for the F25 composite, but was 96.3% (0.08%) lower for F90. For F100, 0% absorption was recorded. The other finding was that F40 exhibited the highest compressive strength (σ_comp_ = 39.46 Mpa). In contrast, F20 depicted the lowest compressive strength (σ_comp_ = 11.13 MPa), which was 71.8% lower than the optimal value recorded in F40. Composites heavily filled with recyclate (F90) and fully filled with recyclate (F100) had strengths of 30.6% (σ_comp_ = 27.38 MPa) and 44.3% (σ_comp_ = 21.99 MPa) lower than F40, respectively. F70 had the highest flexural strength (σ_flex_ = 27.77 Mpa). The other composites demonstrated lower strengths with increasing amounts of recyclate and sand. For F20, the flexural strength value (σ_flex_ = 12.78 MPa) was 53.9% lower than F70. Similarly, for the other composites containing a higher amount of additive, flexural strength gradually decreased. The composites F80 (σ_flex_ = 22.15 MPa), F90 (σ_flex_ = 20.55 MPa), and F100 (σ_flex_ = 20.26 MPa) exhibited flexural strength values that were lower than F70 by 20.2%, 25.9%, and 27.1%, respectively. SEM imaging revealed that the contact zone between the aggregate grains and the bonding phase of the recycled film was consistent with no anomalies, cracks, or voids.

The results from this study conclusively prove that it is possible to produce a suitable binder for building composites from LDPE film waste. Appropriately selected proportions of the recyclate and the filler in the form of sand and gravel aggregate make it possible to obtain a composite with technical parameters similar to or in some cases higher than those of traditional cementitious composites. As such, building composites made from LDPE film waste can effectively be used in construction. Finally, it is also worth mentioning that in this type of composite, the bonding phase can be recycled further by melting and the products can thus take on new forms. The recyclability of construction composites is very important in terms of environmental protection, so the presented methods of producing composites are recommended for implementation in industrial operations.

## Figures and Tables

**Table 1:** Technical parameters of LDPE films taken from material manufacturers’ resources.

Feature	Value
Chemical composition	(CH_2_-CH_2_)n
Volumetric density	0.915–0.920 g/cm^3^
Thermal conductivity	0.32–0.35 W/m-K
Tensile strength	24.7 MPa
Relative elongation at yield stress	>9.1%
Temperature resistance	Below +80 °C

**Table 2:** Technical parameters of sand and gravel aggregate taken from material manufacturers’ resources.

Technical Parameter	Unit	Value/Standard Deviation
Specific density	kg/dm^3^	2.65/±0.1
Volumetric density	kg/dm^3^	1.78/±0.1
Compressive strength	MPa	22/±2
Modulus of elasticity	10^2^ MPa	200/±10%
Absorption	%	2.8/±0.3
Degree of shattering	%	16.0/±2%

**Table 3:** Calculations carried out during iterative component selection for a composite containing 20% recyclate by weight and 80% aggregate by weight.

Component	Amount of Substrate in [g] in the Test Mixture	Substrate Density [g/mL].	Volume in [mL]	Conversion Factor of Ingredient Quantity per 1 m^3^ Concrete Mixture	Amount of Substrate [kg/m^3^] of Concrete Mix	Substrate Density [kg/m^3^]	Component Volume in [m^3^]
LDPE recyclate	77.000	0.92	83.70	3895.14	299.93	920.00	0.3260
Sand and gravel aggregate 0–2 mm	308.000	1.78	173.03	3895.14	1199.70	1780.00	0.6740
SUMA	385.000		256.73	3895.14	1499.63		1.0000

**Table 4:** Recipes of individual mixes in terms of ingredients per 1 m^3^ of composite mix.

Component/Quantity in kg/m^3^	F20	F25	F30	F35	F40	F45	F50	F60	F70	F80	F90	F100
LDPE recyclate	299.93	373.93	451.84	506.37	564.80	576.48	611.54	693.34	759.55	817.98	876.41	927.04
Sand and gravel aggregate 0–2 mm	1199.70	1121.80	1054.29	940.40	847.19	704.59	611.54	462.22	325.52	204.49	97.38	0.00
SUMA	1499.63	1495.73	1506.13	1446.77	1411.99	1281.07	1223.08	1155.56	1085.07	1022.47	973.79	927.04
Density of LDPE recyclate [kg/m^3^]	920.00	920.00	920.00	920.00	920.00	920.00	920.00	920.00	920.00	920.00	920.00	920.00
Aggregate density [kg/m^3^]	1780.00	1780.00	1780.00	1780.00	1780.00	1780.00	1780.00	1780.00	1780.00	1780.00	1780.00	1780.00
Mixture volume [m^3^]	1.00	1.04	1.08	1.08	1.09	1.02	1.01	1.01	1.01	1.00	1.01	1.01
Percentage content of aggregate	20.00	25.00	30.00	35.00	40.00	45.00	50.00	60.00	70.00	80.00	90.00	100.00
Percentage of LDPE recyclate	80.00	75.00	70.00	65.00	60.00	55.00	50.00	40.00	30.00	20.00	10.00	0.00

## Data Availability

The original contribution presented in the study are included in the article, and further inquiries can be directed to the corresponding author.
